# Analysis of Alzheimer’s Disease Based on the Random Neural Network Cluster in fMRI

**DOI:** 10.3389/fninf.2018.00060

**Published:** 2018-09-07

**Authors:** Xia-an Bi, Qin Jiang, Qi Sun, Qing Shu, Yingchao Liu

**Affiliations:** College of Information Science and Engineering, Hunan Normal University, Changsha, China

**Keywords:** random neural network cluster, fMRI, classification, Alzheimer’s disease, functional connectivity

## Abstract

As Alzheimer’s disease (AD) is featured with degeneration and irreversibility, the diagnosis of AD at early stage is important. In recent years, some researchers have tried to apply neural network (NN) to classify AD patients from healthy controls (HC) based on functional MRI (fMRI) data. But most study focus on a single NN and the classification accuracy was not high. Therefore, this paper used the random neural network cluster which was composed of multiple NNs to improve classification performance. Sixty one subjects (25 AD and 36 HC) were acquired from the Alzheimer’s Disease Neuroimaging Initiative (ADNI) dataset. This method not only could be used in the classification, but also could be used for feature selection. Firstly, we chose Elman NN from five types of NNs as the optimal base classifier of random neural network cluster based on the results of feature selection, and the accuracies of the random Elman neural network cluster could reach to 92.31% which was the highest and stable. Then we used the random Elman neural network cluster to select significant features and these features could be used to find out the abnormal regions. Finally, we found out 23 abnormal regions such as the precentral gyrus, the frontal gyrus and supplementary motor area. These results fully show that the random neural network cluster is worthwhile and meaningful for the diagnosis of AD.

## Introduction

Alzheimer’s disease (AD) is degenerative and irreversible which results from the cognitive decline. The progress of AD increases with age and the disease easily triggers the other psychiatric diseases, and eventually causing dementia. In 2006, the number of AD patients is 26.6 million all around the world and the number would be quadruple by 2050. Therefore, it is meaningful for clinician to track its progression and diagnose the disease. Currently, there have been many different neuroimaging techniques which can be applied to diagnose AD, such as ElectroEncephaloGram (EEG) (Engels et al., 2015), Single Photon Emission Computed Tomography (SPECT) (Prosser et al., 2015), Positron Emission Tomography (PET) (Pagani et al., 2017), Magnetoencephalographic (MEG) (Engels et al., 2016), and functional magnetic resonance imaging (fMRI) (Griffanti et al., 2015). Among these techniques, fMRI is widely used in the diagnosis of AD, because it adopts a non-invasive way and could be used to find the differences of brain regions between AD and healthy controls (HC) (Challis et al., 2015).

Machine learning is a method of pattern recognition and has been used for the study of AD in recent years (Moradi et al., 2015; Khazaee et al., 2016). Among many machine learning methods, artificial neural network (ANN) is a useful classification method which evolves from human brain (Er et al., 2016). Several previous studies showed that neural network (NN) was able to be applied to diagnose neurological disease. Suk et al. (2016) proposed a method that combined deep learning and state-space model to classify Mild Cognitive Impairment (MCI) patients from HC, and the accuracy was 72.58%. Gao et al. (2017) employed an advanced convolution neural network (CNN) with 2D and 3D to diagnose AD, and the average accuracy reached to 87.6%. Ortiz et al. (2016) used deep learning architectures to classify AD patients from HC, and the accuracy approximately reached to 90%. Ortiz et al. (2013) used Learning Vector Quantization (LVQ) algorithm to classify AD patients from HC, and the accuracy was close to 90%. Luo et al. (2017) applied CNN to classify AD patients from HC, and the sensitivity and specificity of classification was 1 and 0.93 respectively. Suk et al. (2014) used deep learning to classify AD patients from HC, and the accuracy was close to 93.35%.

In existing studies, a single NN is often used to classify patients with neurological diseases and HC, and the accuracy of classification is considerable which indicates that NN is a powerful classification model (Hirschauer et al., 2015; Anthimopoulos et al., 2016). As the features of neuroimaging data are characterized by high dimension, significant information of original variables would be lost in the process of dimensionality reduction in traditional methods such as principal component analysis, local linear embedding and linear discriminate analysis (Mattioni and Jurs, 2003; McKeown et al., 2007; Mannfolk et al., 2010). In this paper, the method of the random neural network cluster is proposed to classify AD from HC. This method not only could be used in the classification, but also could be used for feature selection. The procedure of this method is as follows. Firstly, we chose Elman NN as the optimal base classifier from five types of neural networks [Back Propagation (BP) NN, Elman NN, PNN, Learning Vector Quantization (LVQ) NN and Competitive NN] based on the results of feature selection, and the accuracies of the random Elman neural network cluster could reach to 92.31% which is the highest and stable. Then we used the random Elman neural network cluster to select significant features and these features could be used to find out the abnormal regions. Finally, we found out 23 abnormal regions, such as the precentral gyrus, the frontal gyrus and supplementary motor area. These results fully show that the random neural network cluster is worthwhile and meaningful for the diagnosis of AD.

## Materials and Methods

### Subjects

The experimental data was collected from the Alzheimer’s Disease Neuroimaging Initiative (ADNI)^1^ dataset which includes a variety of neuroimaging data. The ADNI study was approved by Institutional Review Board (IRB) of each participating site. All ADNI subjects together with their legal representatives should have written informed consent before collecting clinical, genetic and imaging data. The following two criteria need to meet when selecting data. One criterion is that the selected data should be resting-state fMRI data. Another criterion is that the selected data should have mini-mental state examination (MMSE) and clinical dementia rating (CDR) scores, and this criterion ensures that the selected data is homologous. Finally, 61 subjects were selected which consisted of 25 AD patients and 36 HC.

### Data Collection and Preprocessing

Scanning images were acquired on a Philips Medical Systems 3 Tesla MRI scanner. Acquisition parameters included: pulse sequence = GR, *TR* = 3,000 ms, *TE* = 30 ms, matrix = 64^∗^64, slice thickness = 3.3 mm, slice number = 48, flip angle = 80°.

To decrease the influence of signal-to-noise ratio of the fMRI images, the selected data should be preprocessed. The data was preprocessed based on the Data Processing Assistant for Resting-State fMRI (DPARSF) software (Chao-Gan and Yu-Feng, 2010; Wang et al., 2013). The process of the data preprocessing included: converting DICOM format into NIFTI format; removing first 10 time points; slicing timing (Sarraf et al., 2016); realigning (Jenkinson et al., 2002); normalizating images into the echo planar imaging (EPI) template (Misaki et al., 2010); temporal smoothing; removing the effect of low-level (<0.01 HZ) and high-level (>0.08 HZ) noise by a high-pass temporal filtering (Challis et al., 2015); removing covariates such as the whole brain signal and cerebrospinal fluid signal.

### Functional Connectivity of Brain

After the preprocessing steps, the analysis of the functional connectivity of brain was followed. In this paper, we chose functional connectivity as the sample feature. The extraction of the functional connectivity is as follows. Firstly, the images of brain were divided into 90 regions defined by Automated Anatomical Labeling (AAL) brain atlas (Rolls et al., 2015). Secondly, the time series of each region were extracted. Thirdly, the Pearson correlation coefficient between two separated brain regions could be defined as the functional connectivity (Friston et al., 1993). Finally, 4,005 (90 × 89/2) functional connectivity of each subject were taken as their features.

### The Random Neural Network Cluster

The 4,005 functional connectivity belongs to the high-dimensional feature which causes the problems of computation difficulty and dimensions of disaster. Moreover, the high-dimensional features are likely to result in numerous redundant and irrelevant features which may decrease the classification performance. Therefore, the irrelevant features are needed to be removed by feature selection (Azar and Hassanien, 2015).

There are many methods of feature selection, such as principal component analysis, local linear embedding and linear discriminate analysis. However, in these methods, the process of selecting features may cause the loss of the original information, and the classification performance may be unsatisfactory (Zhou et al., 2015; Jolliffe and Cadima, 2016; Alam and Kwon, 2017).

To solve the problem above, this paper proposes the random neural network cluster by randomly selecting samples and features. The random neural network cluster could be used to classify AD patients from HC and select features. In addition, the new method could achieve the purpose of reducing dimensionality, avoid losing significant information and improve classification performance.

### The Design and Classification Accuracy of the Random Neural Network Cluster

**FIGURE 1:**
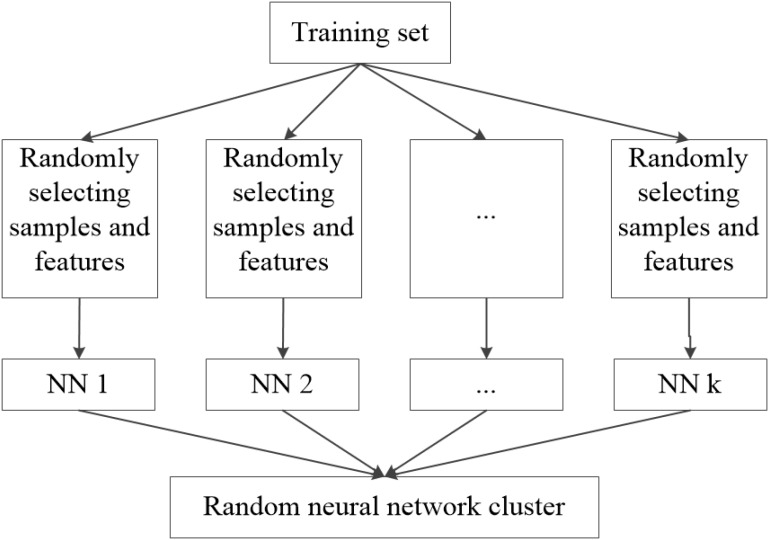
The formation of the random neural network cluster.

The basic thought of the random neural network cluster is ensemble learning whose basic classifier is neural network. The detailed process of establishing the random neural network cluster is as follows. Firstly, the sample dataset *D* is randomly divided into a training set *N*_1_ and a test set *N*_2_, where *D* = *N*_1_ + *N*_2_. The real label of HC and AD is +1 and −1 respectively. Secondly, we randomly select n samples from the training set and m features from the 4,005 features. Thirdly, the selected samples and features are used to establish a single NN and the process of the second and third step is repeated for *k* times. Thus, *k* NNs are obtained which construct the random neural network cluster. It can be seen from this process that the method and the conventional process are essentially not the same in essence. **Figure 1** shows the formation of the random neural network cluster.

When a new sample enters the random neural network cluster, we could obtain *k* class labels from *k* NNs and the majority of class label is made as the predicted label of the new sample. Similarly, we could get the label of each sample in the test set *N*_2_. Then the predicted label is compared with the real label to judge whether they are consistent, and we assume *C* as the number of consistent situations. The accuracy of the random neural network cluster equals to *C*/*N*_2_.

### Extracting Features From the Random Neural Network Cluster

As the features are randomly selected, the NNs constructed by these features have different characteristics. In this paper, the accuracy of each NN is used to evaluate the significance of selected features. The features that make significant contributions to the accuracy of the NN are called the “significant features”. The process of extracting significant features is as follows.

**FIGURE 2:**
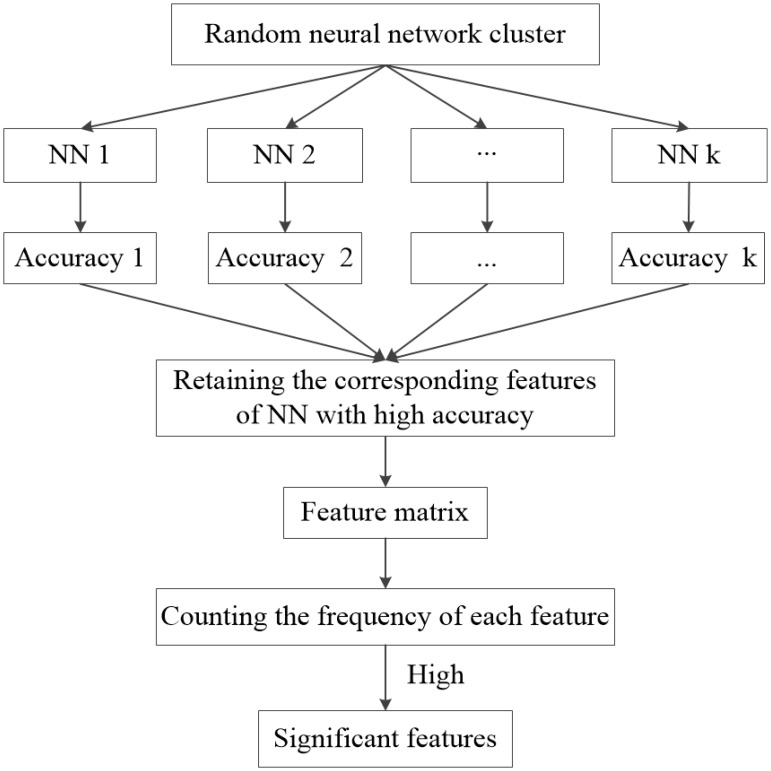
The flow of significant feature extraction.

Firstly, the test samples are used to obtain the accuracy of each NN in the random neural network cluster. If the accuracy of a NN is high, the corresponding features are significant. Next, the features in each NN with high accuracy are extracted to form a feature matrix. Finally, we count the frequency of each feature in the feature matrix, and extract the features with high frequency which are called as “significant features.” **Figure 2** shows the process of selecting “significant features.” The significant features tremendously contribute to the accuracy of a NN, thus they also make great contributions to the accuracy of the random neural network cluster. In this paper, we use the significant features to find the difference between AD patients and HC.

### The Abnormal Brain Regions

As mentioned above, the significant features make great contributions to the accuracy of the random neural network cluster, thus we could find the difference between AD and HC through these significant features. In this paper, the significant features are regarded as the abnormal functional connectivity. As the functional connectivity is defined as the relationship of two brain regions, the extracted significant features could be used to find the abnormal brain regions between AD and HC. In order to estimate the abnormal degree of a brain region, the number of features related to a certain region is considered as the weight. When there is no functional connectivity related to a certain brain region, the weight of the region is 0. When the weight is greater, the abnormal degree of the brain region is higher.

### Experiment Design

The process of the experiment involves six steps in this paper.

Step 1. Building the random neural network cluster. Firstly, 61 subjects are divided into a training set and a test set according to the proportion of 8:2. Thus, the number of training samples and test samples is 48 and 13 respectively. Then, 45 subjects are randomly selected from 48 training samples and 120 features are randomly selected from 4,005 features to build a NN. Similarly, we build 1,000 NNs to construct the random neural network cluster. The result of the random neural network cluster is calculated by using neural network toolbox.

Step 2. Selecting significant NNs. We select the NNs whose accuracy is >0.6 from the 1,000 NNs and call these NNs as significant NNs.

Step 3. Selecting significant features. The features corresponding to the significant NNs form the feature matrix. Then we count the frequency of each feature, and sort the features with a descending order. Finally, we retain the first 240 features which are regarded as the original significant features.

Step 4. Determining the optimal number of the significant features. We change the number of original significant features from 140 to 240 features with a step of 10 as the new significant features, thus there are 11 types of numbers of new significant features. Then we select 120 features from the new significant features to construct a NN. We select 120 features from the original significant features, and change the number of significant features from 140 to 240 features with a step of 10. Thus, we could obtain 11 random neural network clusters. The number of the significant features corresponding to the random neural network cluster with highest accuracy is the optimal number.

Step 5. We repeat the step1-step 4 by using five types of NNs. They are BP NN, Elman NN, PNN, LVQ NN, and Competitive NN. Then we choose one of them as the best base classifier who has the highest accuracy in step 4.

Step 6. Finding abnormal brain regions through the significant features in step 4 based on the best base classifier.

## Results

### The Demographic Information of Participants

In this study, the selected 61 subjects include 25 AD patients and 36 HC. The gender and age difference between the AD group and HC group data are examined by two-sample *t*-test and chi-square test respectively. The result is shown in **Table 1**. It is referred that the two groups have no statistical significance in the gender and the age.

**Table 1:** The information of participants.

Variables (Mean ± *SD*)	AD (*n* = 25)	HC (*n* = 36)	*P*-value
Gender(male/female)	12/13	20/16	0.784
Age	74.59 ± 7.03	77.00 ± 6.61	0.177

*AD, Alzheimer’s disease; HC, Healthy Control.*

### Classification Results

**FIGURE 3:**
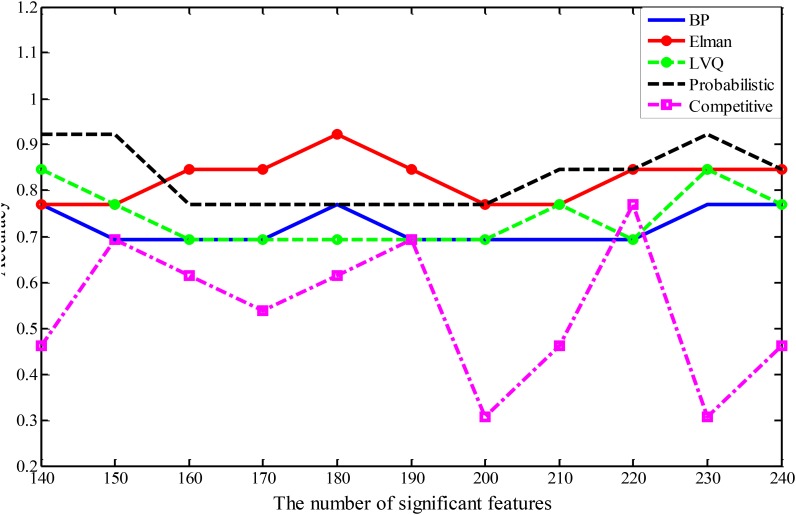
The accuracies of five different types of random neural network clusters.

In this study, five different types of NNs are applied to construct five different types of random neural network clusters. The number of significant features in each random neural network cluster changes from 140 to 240 and the step is 10. Therefore, we can obtain 11 results for each type of random neural network cluster, and their parameters have been made appropriate adjustments to get better results. The accuracies of five types of random neural network clusters are shown in **Figure 3**. It is referred that the accuracies of random Competitive neural network cluster are not stable; the accuracies of random Elman neural network cluster and random Probabilistic neural network cluster are high, and their highest accuracy reaches to 92.31%; the accuracies of random BP neural network cluster and random LVQ neural network cluster are lower than the random Elman neural network cluster. The accuracies of the random neural network cluster changes when the number of features changes. As the highest accuracies of the random Elman neural network cluster are relatively stable and high, this paper chooses Elman neural network as the best base classifier. From **Figure 3** we can learn that when the number of significant features is 180, the accuracy is the highest. Therefore, 180 is the optimal number of significant features.

**FIGURE 4:**
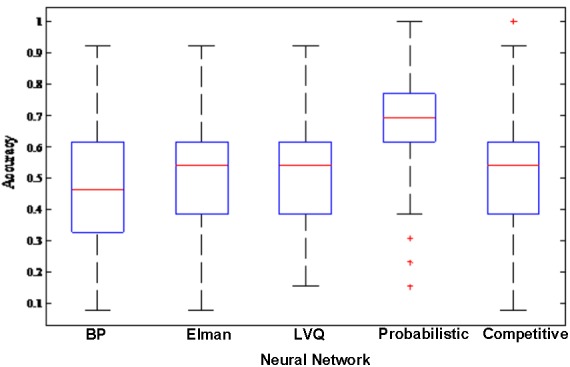
The corresponding 1,000 NNs’ accuracies of five types of random neural network clusters.

In order to better compare the performances of the random neural network cluster and a single NN, we display the corresponding 1,000 NNs’ accuracies of five types of random neural network clusters in the **Figure 4**. From **Figures 3**, **4** we could learn that the accuracy of random neural network cluster is higher and more stable than a single NN except for the random Competitive neural network cluster. In addition, we also show the training errors, test errors and running time of five types of random neural network clusters in **Table 2**.

**Table 2:** The errors and running time of five types of random neural network clusters.

Base classifier (Mean ± *SD*)	Training errors	Test errors	Time (hour)
BP NN	0.2764 ± 0.0375	0.2797 ± 0.0388	2
Probabilistic NN	0.1709 ± 0.0712	0.1678 ± 0.0672	10
Elman NN	0.1782 ± 0.0519	0.1748 ± 0.0497	12
LVQ NN	0.2645 ± 0.0604	0.2587 ± 0.0622	48
Competitive NN	0.4627 ± 0.1532	0.4615 ± 0.1538	18

### The Abnormal Brain Regions

**FIGURE 5:**
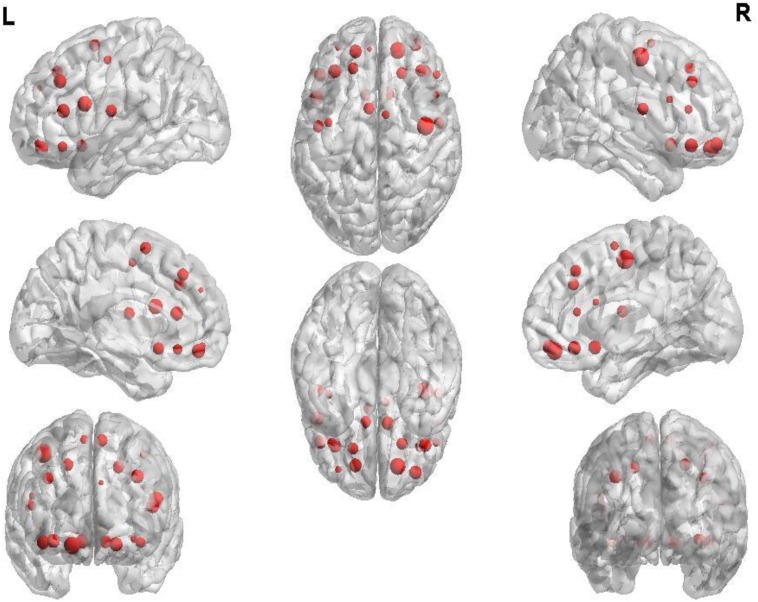
The weight of brain regions.

The first 180 features constitute the optimal feature set which is used to find the abnormal brain regions between AD patients and HC. **Table 3** shows the abnormal brain regions with higher weight, their abbreviation and volume. **Figure 5** shows the abnormal degree of brain regions by using the Brain-NetViewer^2^. The node represents the brain region. The size of a node represents the weight of the brain region and it also indicates the abnormal degree of the brain region.

**Table 3:** Abnormal brain regions.

Regions	Abbreviation	Volume	Weight
The precentral gyrus	PreCG.L	[−39 −6 51]	19
The middle frontal gyrus	MFG.L	[−33 33 35]	
The olfactory cortex	OLF.L	[−8 15 −11]	
The orbital part of superior frontal gyrus	ORBsup.R	[18 48 −14]	18
The triangular part of inferior frontal gyrus	IFGtriang.L	[−46 30 14]	
The supplementary motor area	SMA.L	(−5 5 61)	
The orbital part of superior frontal gyrus	ORBsup.L	[−17 47 −13]	17
The orbital part of middle frontal gyrus	ORBmid.L	[−31 50 −10]	
The precentral gyrus	PreCG.R	[41 −8 52]	16
The dorsolateral of superior frontal gyrus	SFGdor.R	[22 31 44]	
The orbital part of middle frontal gyrus	ORBmid.R	[33 53 −11]	
The orbital part of Inferior frontal gyrus	ORBinf.L	[−36 31 −12]	
The orbital part of Inferior frontal gyrus	ORBinf.R	[41 32 −12]	
The olfactory cortex	OLF.R	[10 16 −11]	
The middle frontal gyrus	MFG.R	[38 33 34]	15
The rolandic operculum	ROL.L	[−47 −8 14]	
The rolandic operculum	ROL.R	[53 −6 15]	
The dorsolateral of superior frontal gyrus	SFGdor.L	[−18 35 42]	14
The opercular part of inferior frontal gyrus	IFGoperc.L	[−48 1319]	
The opercular part of inferior frontal gyrus	IFGoperc.R	[50 15 21]	
The triangular part of inferior frontal gyrus	IFGtriang.R	[50 30 14]	
The supplementary motor area	SMA.R	[9 0 62]	
The medial of superior frontal gyrus	SFGmed.L	[−549 31]	

**FIGURE 6:**
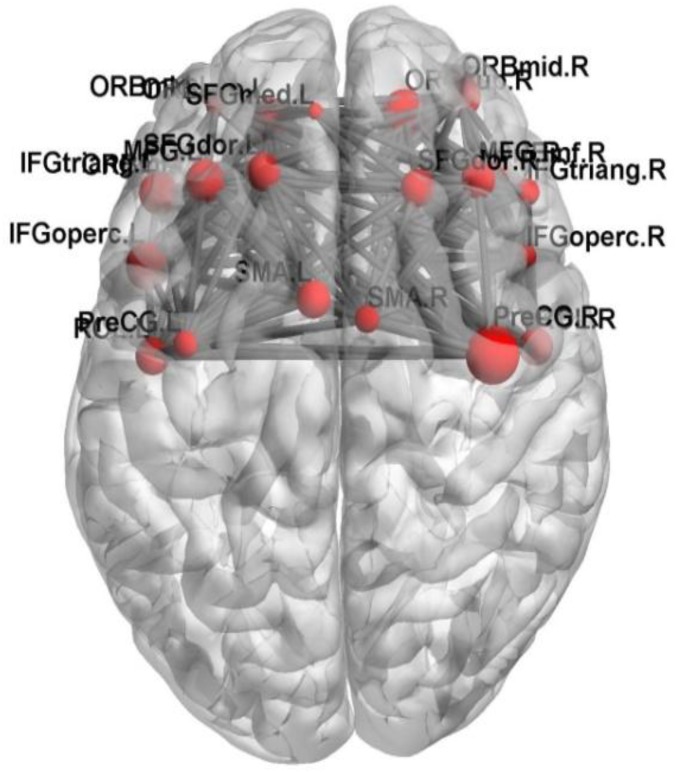
The functional connectivity between the 23 brain regions.

**FIGURE 7:**
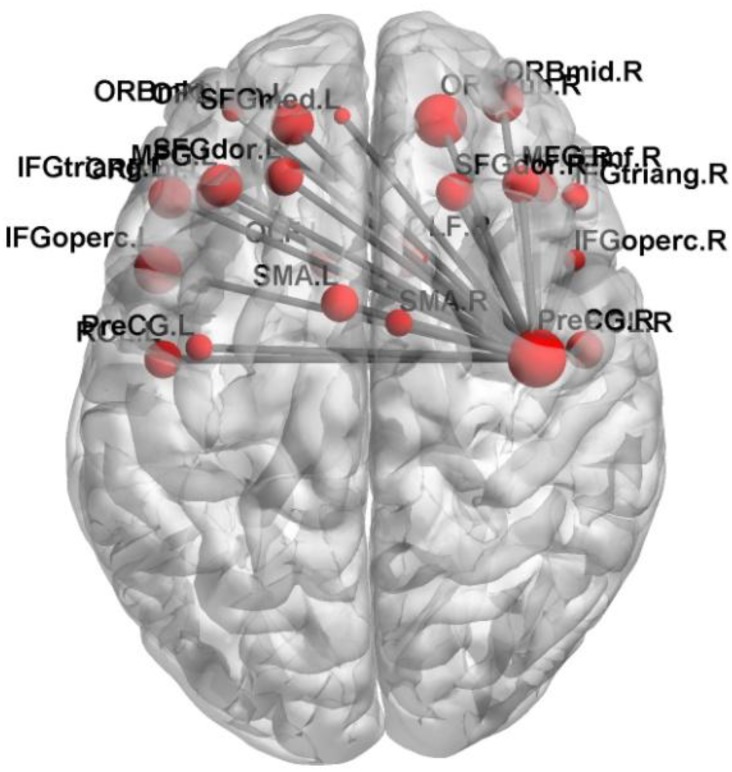
The functional connectivity between PreCG and other brain regions.

In this paper, we focus on the brain region whose weight is >17. **Figure 6** shows the functional connectivity between the 23 brain regions in the optimal feature set. **Figure 7** shows the functional connectivity between PreCG and other brain regions. The node in **Figures 6**, **7** also represents the brain region and the size of a node represents the weight of the brain region. Besides, the line represents the functional connectivity between two brain regions.

## Discussion

### Classification Performance

A number of researchers have tried to classify and diagnose AD patients from HC in the past few years. For instance, Khazaee et al. (2016) applied machine learning methods to classify MCI patients, AD patients and HC, and the accuracy of AD and HC is 72%. Kim et al. (2016) applied DNN to classify schizophrenia (SZ) patients and HC based on fMRI image, and the accuracy is 85.8%. Zhang et al. (2014) proposed a kernel support vector machine decision tree (kSVM-DT) to classify MCI patients, AD patients and HC based on the MRI data, and the accuracy of AD and HC is 96%. However, there were several disadvantages in these studies. For instance, as the features of neuroimaging data are characterized by high dimension, significant information of original variables would be lost in the process of dimensionality reduction in traditional methods.

In this paper, the new method was proposed to avoid the loss of information and improve classification performance. We used five different types of NNs as the base classifier to build five different types of random neural network clusters. In the five types of random neural network clusters, the classification accuracies of the random Elman neural network cluster and the random PNN cluster could reach to 92.3%. As the resting-state fMRI data dynamically changes in a period of time and the Elman NN is able to deal with the time and spatial domain data, the Elman NN is more suitable for the fMRI data. The accuracies of the random Probabilistic neural network cluster could also reach to 92.3%, but the running time of the PNN is long which makes it not suitable for the base classifier of the random neural network cluster. Thus, we finally choose the random Elman neural network cluster as the best base classifier. To solve the problems caused by the high-dimensional data, the traditional methods suffer from the loss of some information in the process of reducing dimensionality. In this paper, we proposed a random neural network cluster which could be a good solution in dealing with the calculation of large samples and high-dimensional data. Moreover, the highest accuracies of the random Elman neural network cluster could reach to 92.3%. Therefore, this method could process high-dimensional data without information loss and improve classification performance.

The basic classifier used in this paper is the neural network. This classifier is indeed an existing classifier, and it is not the innovation point of this paper. The innovation of this article is the integrated cluster, which is the innovation in structure.

This paper researches on fMRI image, there is no innovation in the classification indicators of this article. But the classification indicators have been used in research. We used the random neural network cluster to classify the subjects and feature selection, and we got good results. That is the innovation in application.

### The Additional Details of the Random Neural Network Cluster

In the part of method, we have introduced many elementary details in our method and in this part we would introduce some additional details.

In order to make the random neural network cluster less complex, each NN occupies the same weight in the random neural network cluster. When determining the label of a new sample, each NN in the random neural network cluster predicts the label of the sample. The majority of class label is made as the predicted label of the sample which is equivalent to that the weight of each NN is the same. From **Figures 3**, **4** we could learn that the accuracy of a single NN is lower than the accuracy of the random neural network cluster. Moreover, when the number of significant features changes, the accuracies of five types of random neural network clusters are high and stable except for the random Competitive neural network cluster. This fully demonstrates that the robustness of the random neural network cluster is good.

In terms of the complexity of random neural network cluster, it is mainly reflected in the following two aspects. One aspect is that the number of base classifiers is large which makes the process of constructing a random neural network cluster complex. Another aspect is that we need to construct multiple random neural network clusters during finding the optimal feature set which also makes the method complex.

There are two types of parameters in our method. One type is the parameters of the random neural network cluster which are decided by the accuracy of the random neural network cluster through hundreds of experiments. Another type is the parameters of the NN which are decided by the neural network toolbox that automatically selects the optimal parameters.

The random neural network cluster is constructed by the training set and the performance is tested by the test set. The experimental results show that the random neural network cluster not only performs well on the training set, but also performs well on the test set. This fully demonstrates that the overfitting does not exist in the random neural network cluster.

### Abnormal Brain Region Analysis

The functional connectivity differences between AD and HC could be used to find out the abnormal brain regions and we finally detected 23 abnormal brain regions between AD and HC. They are the PreCG, the OLF, the ORBsup, the IFGtriang, SMA, the SFGdor, the ORBmid, the ORBinf, the SFGmed.L, the IFGoperc, the ROL, and the MFG.

Previous studies have concluded that these abnormal brain regions are associated with AD patients. Khazaee et al. (2015) found that the lingual gyrus, the occipital gyrus and the superior frontal gyrus are abnormal brain regions in AD patients. Agosta et al. (2012) pointed out that the functional connectivity changed in the default mode network (DMN) and frontal networks in AD patients. Wang et al. (2007) discovered the decreased abnormal functional connectivity in the prefrontal and parietal lobes, meanwhile the increased abnormal functional connectivity in the occipital lobe in AD patients. Greicius et al. (2004) found that AD patients showed less deactivation in the anterior frontal, precuneus and posterior cingulate cortex. He et al. (2007) concluded that regional coherence of AD patients significantly decreased in the posterior cingulate cortex/precuneus. Binnewijzend et al. (2012) declared that the abnormal brain regions of AD patients located in the precuneus and posterior cingulate cortex. Golby et al. (2005) concluded that impaired activation changed in the temporal lobe and fusiform regions in AD patients. Grossman et al. (2003) found the activation of AD patients in the left poster lateral temporal and inferior parietal cortex. It is referred that our results are consistent with previous studies. In this paper, we focus on the precentral gyrus and the frontal gyrus which have larger weights.

#### Precentral Gyrus (PreCG)

The PreCG has the greatest weight in the abnormal brain regions. It is referred that the PreCG makes a great contribution to classify AD and HC in the random Elman neural network cluster. The PreCG locates in the primary motor area (Hopkins et al., 2017), and the superior part of PreCG is responsible for motor hand function (Yousry et al., 1997; Nebel et al., 2014).

Existing studies have found that the PreCG is abnormal in AD patients. Kang et al. (2013) showed that AD patients performed significant cortical thinning in superior and medial frontal gyrus, left precentral gyrus, postcentral gyrus, paracentral lobule, precuneus and superior parietal lobule. Bi et al. (2018) proposed a method of random support vector machine cluster to diagnose AD, and found out several disorder regions including inferior frontal gyrus, superior frontal gyrus, precentral gyrus and cingulate cortex. Zhang et al. (2015) pointed out that the middle occipital gyrus, the postcentral gyrus, the PreCG and precuneus were important in diagnosing AD. Brickman et al. (2015) discovered that the PreCG and right middle frontal gyrus were abnormal in AD patients based on T2-weighted MRI. Dani et al. (2017) used white matter hypointensity (WMH) volume to diagnose AD based on MRI and PET data, and they found out the difference between the PreCG, and the right medial and anterior part of orbital gyrus.

The abnormal functional connectivity between PreCG and other brain regions may lead to the physical movement dysfunction in AD patients. The above results revealed that PreCG may be a clinical diagnosis of AD in future.

#### Frontal Gyrus (FG)

The FG had a relatively higher weight in the abnormal regions. It is referred that the FG makes a great contribution to classify AD and HC in the random Elman neural network cluster. The left of inferior frontal gyrus is associated with language (Costafreda et al., 2006). The superior frontal gyrus contributes to higher cognitive functions and particularly the learning and working memory (WM) (Boisgueheneuc et al., 2006; Carter et al., 2006). Eliasova et al. (2014) explored that the improvement of attention and psychomotor speed resulted from the abnormity of the right IFG in AD patients.

Existing studies have found that the FG is abnormal in AD patients. Schultz et al. (2015) found out several abnormal brain regions in AD patients including the hippocampus, the posterior cingulate, the anterior cingulate and the middle frontal gyrus. Griffanti et al. (2016) pointed out that there was difference in orbito frontal network between AD patient and HC. Yetkin et al. (2006) evaluated brain activation in AD patients and HC based on fMRI while performing a WM task and found that AD group showed more activation in the right superior frontal gyrus, bilateral middle temporal, middle frontal, anterior cingulate and fusiform gyri. Karas et al. (2007) used voxel-based morphometry (VBM) to examine AD patients, and they found out several disorder regions including the left superior and inferior temporal gyrus, and the left superior frontal gyrus. Our experiment results are consistent with the existing studies.

The abnormal functional connectivity between FG and other brain regions may lead to the memory dysfunction in AD patients. This abnormal brain region has significant effect on the identification of potentially effective biomarkers for the diagnosis of AD.

In this paper, the random neural network cluster is proposed to classify AD patients and HC and the abnormal brain regions are found out on the basis of fMRI data. Moreover, these findings suggest that the random neural network cluster might be an appropriate approach for diagnosing AD. This new method has some advantages. Firstly, the NNs make different contributions to the random neural network cluster which could make full use of each NN’s characteristics, thus it could avoid losing information. Secondly, it is able to effectively deal with a large dataset even when there is many missing data. Finally, it is also able to select significant features from high-dimensional features. The new method presents a good classification performance with accuracy of 92.3% and detects several abnormal brain regions which would have influence in diagnosing AD. However, it has several limitations. Firstly, this paper only used the fMRI data and the future studies could integrate other imaging data to obtain comprehensive brain activity. Secondly, this paper only studied the brain activity, and the future studies could combine the brain and cerebellum activity. Thirdly, this paper only studies the brain difference between AD patients and HC, and the future studies could focus on the brain relationship in AD patients. Finally, the functional connectivity was regarded as feature in this paper, and the future studies could choose other attributions of the brain as feature such as clustering coefficient, degree and shortest path.

## Ethics Statement

This study was carried out in accordance with the recommendations of National Institute of Aging-Alzheimer’s Association (NIA-AA) workgroup guidelines, Institutional Review Board (IRB). The study was approved by Institutional Review Board (IRB) of each participating site, including the Banner Alzheimer’s Institute, and was conducted in accordance with Federal Regulations, the Internal Conference on Harmonization (ICH), and Good Clinical Practices (GCP).

## Author Contributions

X-aB proposed the design of the work and revised it critically for important intellectual content. QSu and QJ carried out the experiment for the work and drafted part of the work. YL and QSh collected, interpreted the data, and drafted part of the work. All the authors approved the final version to be published and agreed to be accountable for all aspects of the work in ensuring that questions related to the accuracy or integrity of any part of the work are appropriately investigated and resolved.

## Conflict of Interest Statement

The authors declare that the research was conducted in the absence of any commercial or financial relationships that could be construed as a potential conflict of interest.

## Footnotes

1 http://adni.loni.usc.edu/

2 http://www.nitrc.org/projects/bnv/
